# What electrodes can be used to measure mu rhythm (de)synchronization in the context of speech comprehension studies? An insight from theoretical analysis

**DOI:** 10.3389/fnhum.2026.1676434

**Published:** 2026-02-03

**Authors:** Dmitry A. Chegodaev, Polina A. Pavlova, Natalia Karpova

**Affiliations:** 1 Sirius University of Science and Technology, Krasnodar, Russia; 2 Laboratory for Brain and Neurocognitive Development, Ural Institute of Humanities, Ural Federal University named after the first President B. N. Yeltsin, Yekaterinburg, Russia

**Keywords:** EEG, mirror neuron system, mu desynchronization, mu rhythm, sensorimotor integration, speech comprehension

## Introduction

The electroencephalographic (EEG) mu rhythm, or sensorimotor rhythm, is an oscillatory activity that consists of two nonharmonic components in the alpha (8–13 Hz) and beta (~13–30 Hz) frequency ranges. It is considered a neural marker for studying sensorimotor integration during speech perception and comprehension tasks (Saltuklaroglu et al., 2018; Inamoto et al., 2023).

Mu rhythm suppression (or desynchronization) is registered primarily over the sensorimotor cortex and serves as an indirect indicator of activity of mirror neuron system (MNS), which is a key neural network mediating action observation/execution and speech processing (Hickok, 2009; Gatti et al., 2017). However, the mu rhythm, as well as its suppression, is not limited exclusively to sensorimotor regions of the brain.

As more attention is being paid to mu rhythm reactivity as a marker of speech comprehension, one principal aspect of research is to select the optimal EEG electrodes for capturing mu activity.

A substantial body of research that investigated the linkage between speech processing and mu rhythm reactivity was performed on 10–20 systems and typically considered central (C3, C4, and corresponding sites in high-density systems), parietal (P3, P4, Pz), and frontal (F3, F4, Fz) electrodes as regions of interest (Cuellar et al., 2012; Antognini and Daum, 2019; Patzwald et al., 2020; Mikhailova et al., 2021; Salo et al., 2023). Whereas the selection of temporal sites, particularly T3, T4, T5, and T6, has been reported only in limited studies (Belalov et al., 2020).

The relatively poor spatial resolution of scalp-recorded EEG complicates accurate localization of mu rhythm signals and their isolation from other oscillatory activity (e.g., occipital alpha rhythm). Besides, speech comprehension tasks suggest evaluating the activity of widely represented cortical networks, including temporal, parietal, and frontal areas, requiring a search for methodologies to define optimal sets of electrodes.

This paper aims to shape the arguments and create a comprehensive rationale behind electrode selection in high-density EEG systems for mu rhythm research in speech perception studies.

The manuscript is organized in several concise sections that together focus on the interrelation between specific Brodmann areas (BAs), their certain cytoarchitectonic characteristics, and the neurotransmitter systems involved in mu rhythm generation as well as MNS activity and speech processing, thus providing the theoretical support for the strategy of electrode selection. The results of our analysis led to the identification of the temporo-parietal electrode cluster of electrodes (over BA22) as the most optimal for measuring mu reactivity in speech comprehension tasks, whereas frontal, central, and part of temporo-parietal clusters were assigned auxiliary roles.

## Dominant cortical substrates of speech comprehension

Reviewing and highlighting the brain regions (BAs) crucially associated with language comprehension in this section is the first step in building a rationale for EEG electrode selection.

Certain aspects of the physiological roles of the canonical cortical language areas and their anatomo-functional interactions remain a subject of debate.

Wernicke's area (mainly BA 22, BA 21, BA 41, and BA 42) has long been postulated to have the highest priority on speech comprehension. At present, additional BAs (BA20, BA37, BA38, BA39, and BA40) are considered to be a part of the so-called “extended Wernicke's area” (Ardila et al., 2016).

There is a compelling reason to believe that the classical concept positing Broca's area (BA44, BA45) as an exclusively motor speech center is too simplistic. Together with supplying motor speech, this region is responsible for various functions in language comprehension. For instance, there is data indicating that the inferior frontal gyrus (IFG) pars opercularis (BA44) and especially pars triangularis (BA45), parts of Broca's area, are heavily implicated in processing syntactic information and semantic analysis (Liuzzi et al., 2024). Thus, the “classical” roles of Wernicke's and Broca's areas are not isolated; rather, they function as interrelated high-order hubs within a broader linguistic system.

This integrated view is formalized in contemporary neurobiological models of language. A widely accepted dual-stream model of speech and language processing proposed by Hickok and Poeppel posits that sensoryinformation is routed into two distinct but interacting neural pathways (Hickok, 2022). Within the dual-stream model, the temporo-frontal extreme capsule fasciculus (TFexcF) constitutes the ventral stream of language processing. It is involved in the mapping of auditory speech signals into conceptual and semantic representations, thus primarily contributing to speech comprehension (López-Barroso and de Diego-Balaguer, 2017; Weiller et al., 2021; Barbeau et al., 2024). TFexcF represents the anatomical connection between the pars triangularis (i.e., BA45) and the superior temporal sulcus (BA21, BA22) and the medial temporal gyrus, which is primarily associated with BA22 (Barbeau et al., 2024).

Anatomically, the dorsal network comprises suprasylvian projections (primarily arcuate fasciculus) between the posterior superior temporal/inferior parietal (roughly BA40 and BA22 to some extent) and inferior frontal gyrus (BA44/45) and premotor cortex (BA6) (Ries et al., 2019). But limited research reports that cortical terminations of the arcuate fasciculus reach BA44, BA45, BA46, BA47, BA6, and BA9 in the frontal lobe (Rilling et al., 2008).

While the dorsal stream is predominantly involved in speech production, its activity is considered most important for speech perception during the language acquisition phase. It's also thought to support sensorimotor integration for auditory sequences and supply syntactic processing (Rauschecker, 2011; Ries et al., 2019). This significantly blurs the line of a strict functional separation between motor and sensory speech systems and suggests that language comprehension relies substantially on the dorsal stream in addition to the ventral network. In this regard, there is an intriguing paper by Ono et al. that demonstrated the bidirectional neural activity between Broca's and Wernicke's areas during interactive verbal communication in listeners, whereas speakers were characterized by only a unidirectional relationship (Ono et al., 2022).

The BAs initially identified in this section (and summarized in Table 1) represent basic anatomical components, each a potential target for selection strategy. Table 1 could serve as a central source of reference that contains information about the rationale discussed in more details in the following sections.

**Table 1:** Domains of evidence supporting the rationale behind the strategy of electrode selection.

**Main BAs**	**Relation to the linguistic system**	**Relation to the MNS**	**Cytoarchitectonic features**	**Dopaminergic and oxytocinergic actions**	**Electrodes (Scrivener and Reader, 2022)**	**Electrodes (Koessler et al., 2009)**
9 (9/46)	Part of the dorsal stream	A well-established component of MNS	Prominent layer 3	High D1 receptor density; extensive dopamine projections	AF3, AF4, AFz, F3, F4, F5, F6	AF3, AF4, AFz, AF6
21	Wernicke's area (extended); part of the ventral stream	The area is related to the MNS	Considerable layer 3 and wide layer 5	High D2 receptor density; undergoes oxytocin modulation	T7, TP7, FT10	C3, T7, TP7, TP8
22	Wernicke's area (core); part of the ventral stream	A well-established component of MNS	Prominent layer 3 and layer 5	High D2 receptor density; expression of D1 mRNA; undergoes oxytocin modulation	T8	CP5, FT7, FT8
39	Wernicke's area (extended)	The area is related to the MNS	Considerable layer 3 and layer 5	High D1 receptor density; undergoes oxytocin modulation	P5, P6, CP5, CP6	P3, P4, P5, P6
40	Wernicke's area (extended); part of the dorsal stream	A key region of the human MNS (core)	Considerable layer 3 and layer 5	High D1 receptor density; undergoes oxytocin modulation	CP3, C5, C6	CP3, CP4, CP6
44 (44/6)	Broca's area (pars opercularis); part of the dorsal stream	A key region of the human MNS (core)	Prominent layer 3 and layer 5	Undergoes significant dopamine modulation	FT7	No data
45	Broca's area (pars triangularis); part of the dorsal and ventral stream	A well-established component of MNS	Prominent layer 3	Undergoes dopamine and oxytocin modulation	F7, F8	F7, F8

The table shows a set of criteria with different priority levels was established to guide the selection of cortical areas and their corresponding EEG electrodes. The anatomo-functional criterion (called “relation to the linguistic system”) has been determined as the primary and highest priority criterion. This prioritization was driven by two factors: (1) the nature of the issue under investigation and (2) the relatively localized distribution of these anatomic substrates. The remaining domains are regarded as lower-order criteria that provide supportive justification for the selection. Following the reasoning outlined in the sections below, the severity of the BAs' mirror properties has been defined as the most important lower-order criterion, while neurochemical profile was assigned lesser importance.

Thus, the dual-stream model by Hickok and Poeppel indicates that speech comprehension is mediated by the coordinated work of both ventral (including Wernicke's extended area) and dorsal (including Broca's area) processing networks. Keeping this in mind, and given the (1) close anatomical proximity of mirror neurons to cortical language areas, (2) strong association of the dorsal stream with sensorimotor integration, there is a substantial functional link that might reasonably be expected between the dorsal and ventral networks and the MNS (described in more details below).

## Implication of mirror neuron system in speech comprehension

There is a lack of consensus within the scientific community regarding whether mu rhythm suppression reliably reflects the functioning of the MNS.

However, since sensorimotor (mu) rhythm is still considered a potential index of MNS activity, the identification of cortical areas integral to both the MNS and the language network could provide an important part of a theoretical basis for our strategy, emphasizing the focus on language-relevant BAs discussed in the last section.

That is, in this section we try to prove that areas with “mirror” properties can be considered as nodes where language comprehension and sensorimotor integration (indexed by the mu rhythm) converge.

Regions with mirror properties might be involved in simulating the action during understanding of action-related language, which is discussed in literature under the term “embodied language processing” (Hoedemaker and Gordon, 2014; Tian et al., 2020). At present the relevance of this concept has not diminished; rather, it has intensified. For instance, mental imagery of words with a motor component is thought to be implicated in the comprehension of figurative speech (Garello et al., 2024).

Consequently, the embodied view of language comprehension could provide a conceptual link between the functional properties of the MNS and the cognitive processes underlying speech comprehension.

According to Pineda (2008) three cortical areas (i.e., inferior frontal gyrus, inferior parietal lobule, and superior temporal sulcus) could be conceptualized as the “core” of the MNS in humans. Similar to this view, but more specifically, the meta-analysis by Molenberghs et al. (2012) identified BA44, BA7, BA9, BA6, BA40, and, to a large extent, BA22, BA45 as the most frequently reported lateral cortical areas (BAs) exhibiting mirror neuron activity. As discussed above, BA22, BA40, BA44, and BA45 are considered the cortical areas directly involved in speech comprehension, whereas the involvement of BA9 and BA6 in this function is not as obvious. Nevertheless, there is sufficient evidence supporting the importance of BA9 for strategic inference processes during language comprehension (Chow et al., 2008). And BA9/46 is regarded as critical for understanding idiomatic expression (Sela et al., 2012).

Neuroimaging studies provide substantial support for the centrality of BA6, 44, 9, 46, and 40 in working memory processing (Cabeza and Nyberg, 2000; Ramsey et al., 2004). Scientific literature emphasizes the critical role of working memory in language comprehension by maintaining semantic and phonological information (for example, see Martin, 2021). In this context it is important to note that MNS not only mediates action-related semantics but is also known to contribute to phonological working memory (Jairew et al., 2025).

Despite the fact that human BA6 is traditionally thought of as a “motor” area, data are gradually being collected indicating its involvement in speech understanding. Distinct neuroimaging studies demonstrate that motor areas, namely BA4a/6, are activated by listening to speech (for example, Wilson et al., 2004). Likewise, available literature discussed the role of the ventral part of BA6 in phonological processing (Hagoort, 2005).

Thus, a significant portion of the Brodmann areas within the MNS overlaps with the regions that form the main cortical substrates for speech comprehension.

Altogether, these arguments support the belief that MNS is fundamentally integrated with the language comprehension network, and assessing mirror activity with mu rhythm over the identified Bas could indirectly estimate the contribution of MNS to speech perception and comprehension processes.

## Cytoarchitectonic features of cortices involved in speech processing and mu rhythm generation

It is generally accepted that mu/alpha activity relies heavily on the laminar architecture of the cortex (i.e., mu activity seems to be significantly layer-specific). Therefore, in the context of our discussion, regional cytoarchitectonic differences can be employed as a filter to guide the selection of electrodes.

Although multiple cortical layers contribute to EEG signals, layer (L) 5 pyramidal neurons play a major role due to their size and the perpendicular orientation of their apical dendrites to the cortical surface (Kirschstein and Köhling, 2009). Likewise, neurons of layer 5 are considered to be critical for the generation of alpha/mu activity (Haegens et al., 2015; Scheeringa et al., 2016), especially given their extensive connections with thalamic nuclei. However, the significance of supragranular cortical layers for alpha activity is still subject to discussion (Scheeringa et al., 2016). Particular emphasis in this regard is given to L3 by reason that it contains a significant number of pyramidal cells and the thalamus sending strong projections to this layer of the cortex (especially primary motor and somatosensory cortices). Since mu rhythm is a thalamocortical phenomenon and considering that pyramidal cells in L3 can modulate firing of L5 neurons, activity of L3 neurons at least contributes to the alpha/mu-range oscillations.

L3 pyramidal neurons form horizontal excitatory connections between different cortical regions and mediate higher-level cognitive functions, including speech (Larsen et al., 2022). For instance, a study performed by Moerel et al. (2019) illustrates that neuronal populations in superficial layers of primary auditory cortex displayed an increased complexity of sound processing but were characterized by slower responses than neurons of L4. It was therefore suggested that primary auditory cortex supply complex auditory processing in humans together with physical sound analysis and indicate the importance of L3 neurons for processes underlying speech understanding. A paper authored by Zachlod et al. (2020) illustrates that BA22 is characterized by dense layers 5 and 2/3 (in the upper bank of the superior temporal sulcus – STS1) and well-defined large pyramidal cells in L3 in the temporal area Te3 – a posterior part of BA22. The BAs 20 and 21 have a smaller layer 3 than BA22 but a wide L5. L3 and L5 are fairly noticeable in BA39 and BA40 but less prominent than in BA22 and L5 of BA40 is smaller than that of BA39 (Zilles, 2003).

The thickest L3 among all BAs is observed in BA10, which is only indirectly (through the maintaining of working memory) involved in speech perception (Burgess and Wu, 2013). L3 in BA10 consists of large pyramidal neurons, and it has an extensive dendritic branching network, highlighting its role in higher-order (associative) functions. Together with BA10, adjacent BA9 also has well-developed L3 with large pyramidal cells and is characterized by extensive associative projections (Prkačin et al., 2024). Interestingly, BA4, which corresponds to the part of the central electrodes, is characterized by prominent L3 and 5, comprising together 70% of the cortical thickness of this area (Alan et al., 2023).

BA44 is a dysgranular area characterized by large pyramidal cells in L3 and in L5. BA45 differs from BA44 by the presence of a well-developed L4 and strikingly large pyramidal neurons in the deeper part of L3 (Petrides, 2005).

Thus, it may be concluded that cytoarchitectonic features of MNS- and language-related BAs, namely the thickness of L3 and/or L5, can also guide the selection of BAs and associated electrodes as appropriate for capturing mu activity during a specific task.

## Linking the dopamine and oxytocin neurotransmitter systems to mu range activity and speech comprehension

This section represents an additional justification for strengthening the logical framework of our conception by delineating the contributions of specific neurotransmitter systems supporting speech and language processing and underlying the modulation (rather than generation) of mu activity. It should be noted that lines of reasoning provided here are highly speculative in nature, because no hard evidence has been provided yet to determine a comprehensive picture of the exact extent of different neurotransmitters' involvement in the processes of mu rhythm generation and modulation.

Although glutamatergic and gamma-aminobutyric acid (GABA)-ergic systems are major contributors to the mu-rhythm generation, in this section we concentrated on two other neurotransmitter systems: dopaminergic and oxytocinergic. This is due to several reasons: (1) both systems influence the mu range activity, (2) the dopaminergic and oxytocinergic systems are interconnected (for example, see Rappeneau and Díaz, 2024), (3) representation of these systems in the lateral cortex is more localized than glutamatergic and GABA-ergic, (4) dopamine and oxytocin (OXT) systems are linked with MNS (Liu et al., 2025), and (5) dopamine and OXT contribute to speech comprehension (Ye et al., 2017; Cardin et al., 2020).

The existing evidence delineates discernible and robust linkage between the language network (especially dorsal) and the dopaminergic system. There is interesting data from a Parkinson's disease (PD) studydemonstrating more pronounced impairment of comprehension of action-related words (i.e., embodied semantics) in PD patients than non-action words, implying that this specific deficit could be a consequence of the disruption of the dopaminergic pathways to the sensory-motor cortex (Fernandino et al., 2013).

Dopamine-releasing neurons from the substantia nigra establish extensive synaptic connections with cortical neurons of the dorsolateral prefrontal cortex, primarily BA 47 and BA 9/46 (Zhou et al., 2024). In addition, there is limited data indicating that the inferior frontal junction (BA 44/6) is related to dopamine synthesis (Klostermann et al., 2012). The study by Jang et al. could serve as an illustrative example, highlighting the interrelationship between the nigrostriatal tract and the fasciculus arcuatus and the role of the dopaminergic system in the neurochemical foundations of speech (Jang et al., 2023).

It is widely acknowledged that dopamine signaling plays a pivotal role in the functioning of the working memory/attentional system, which is crucial for language, especially phonological and semantic processing (Obermeyer et al., 2022; Matzel and Sauce, 2023).

A study using a high-affinity dopamine D2/D3 receptor tracer performed by Aalto et al. demonstrated increased dopamine release in BA45 and 44 in both hemispheres and ventral parts of BA46 in the left hemisphere, but not in parietal areas (Aalto et al., 2005). Palomero-Gallagher et al. synthesized available data from published research on the distribution of neurotransmitter receptors in the human brain, concluding that D1 receptor densities are relatively high in several regions of the lateral cortex: BA46, BA7, BA39, BA 40, and BA17 (Palomero-Gallagher et al., 2015). These findings are largely concordant with some earlier studies. For instance, the paper authored by Dawson et al. reports about the high density of D1 receptors in L5a of the prefrontal (BA9 area) cortex in humans (Dawson et al., 1987). Another study indicated a high expression of dopamine receptor mRNAs in the prefrontal (BA9, BA11) and temporal neocortex (BA20, BA22) with relative enrichment of D1 mRNA in deeper layers (Meador-Woodruff et al., 1996).

Similarly, animal experimental studies demonstrate that cells containing D1 receptors are located mainly in L5 of the primary motor cortex (also known as BA4) and the prefrontal and orbitofrontal cortex (includes BA10), while the majority of D2 receptor-expressing neurons were observed in L 2/3 of these cortices (Wei et al., 2018; Cieslak et al., 2024). Goldsmith and Joyce in their study, have identified areas 22/42 and BA20 and 21 as regions with high density of D2 receptors (Goldsmith and Joyce, 1996).

That is, the relatively high expression of dopamine receptors in the MNS regions associated with language comprehension further supports the role of the dopaminergic system in the functioning of both neural networks (i.e., the MNS and language networks).

Moreover, dopamine-mediated mechanisms appear to be directly involved in the regulation of the GABA-ergic system. Limited evidence suggests that this process is differently modulated by distinct types of dopamine receptors in the prefrontal cortex: activation of D1 receptors (presumably in L5) increases inhibitory activity, whereas D2 (in L 2/3) receptor activation decreases GABAergic inhibition through separate signaling pathways (for more details, see Trantham-Davidson et al., 2004). These are the pathways potentially contributing to the event-related desynchronization/synchronization of sensorimotor oscillations, knowing that changes in synaptic GABA concentration significantly influence sensorimotor (β-) rhythm power (Muthukumaraswamy et al., 2013). It should be noted, however, that for alpha/mu frequencies, the effect of GABA levels leads to less pronounced changes of power (Groth et al., 2021).

Laminar distribution of dopamine receptors and their influence on GABA inhibition is also consistent with the proposition that L5 is essential for mu rhythm generation, whereas L3 is more important to its modulation, concretely—mu suppression.

The consensus is that oxytocin is a neuropeptide hormone tightly linked to social cognition. Given this role, it is consistent that research implicates oxytocin in modulating the perception of social-emotional cues in speech (Vogt et al., 2023). Experimental evidence points to the fact that OXT plays a significant role in modulating neuronal activity. For instance, OXT influences GABAergic control of mPFC (medial prefrontal cortex) activity (Triana-Del Rio et al., 2022). It is also known that OXT can modulate dopamine neuron excitability (Xiao et al., 2018). Interestingly, layers II/III and V contain apparently greater amount of OXT receptors than other layers in mammalian neocortex (Duchemin et al., 2017). Finally, OXT has been shown to reduce EEG activity in the alpha/mu (8–10 Hz) and beta (15–25 Hz) ranges, suggesting the possibility of enhancing the activity of mirror neurons by OXT (Perry et al., 2010). The oxytocinergic system is tightly linked with the amygdala-hippocampal complex, orbitofrontal cortex and there are direct axonal projections from oxytocin-producing neurons to the medial prefrontal cortex (Alaerts et al., 2019; Raam, 2020). There are limited data between interaction of oxytocin system and lateral cortex. However, some reports indicate that OXT can relatively selectively enhance activity in regions of the lateral cortex, including the superior temporal sulcus (BA21, BA22), inferior frontal gyrus (IFG) pars triangularis (BA45) and inferior parietal lobule (BA39, BA40) and premotor cortex (Riem et al., 2011; Gordon et al., 2013). Oxytocin has also been shown to modulate mirror neuron activity, especially in the context of social interaction (Palmieri et al., 2021).

Thus, the dopaminergic and oxytocinergic systems can modulate activity in the mu range over the regions engaged in speech comprehension, and the mechanism of this modulation appears to be layer-dependent. The high expression levels of dopamine and oxytocin receptors in L3 and L5 further support the rationale for selecting these areas as sources of the mu-rhythm, thereby enhancing our strategic approach to electrode selection.

In the context of our work, this line of argument serves as an important hypothesis-generating element. Nevertheless, empirical work is needed to confirm whether the discussed neurotransmitter systems are indeed significantly related to mu reactivity patterns over the suggested sites.

## Conclusion

In order to suggest optimal electrodes for measurement of mu reactivity within speech comprehension studies, we devised an argumentation strategy derived from several theoretical assumptions. First of all, to delineate the relevant cortical areas (BAs), a set of selection criteria has been formed (listed in order of importance). (1) The BA is a component of the dual-stream model of speech processing (with high priority to the ventral stream), and it has an established role in speech comprehension. (2) The BA has been identified as a cortical region exhibiting mirror properties in humans. (3) The BA possesses a well- developed cortical layer V and III, which are considered to be critical for the generation and modulation of thalamocortical oscillations in the mu range. (4) BA shows a significant presence of dopaminergic and oxytocinergic signaling systems involved in mu-rhythm reactivity.

Secondly, to transition from cortical regions to EEG recording sites, we employed data from probabilistic stereotactic mapping studies. Specifically, we utilized the findings of the studies byKoessler et al. and Scrivener and Reader, which provide the variability of EEG site positions and their underlying brain regions (Koessler et al., 2009; Scrivener and Reader, 2022). An electrode was considered a candidate for inclusion if it was associated with one of our pre-identified BAs (summarized in Table 1).

Considering that selected BAs do not equally satisfy the criteria, tentative and speculative stratification may be suggested for the candidate electrodes.

In our view, CP5, FT7, FT8, and T8 (presumably T7 and CP6, since they are homologous to T8 and CP5, respectively) can be recommended as a first-line choice because this choice completely aligns with the discussed strategy. Specifically: (1) BA22 is a core of Wernicke's area and an integral part (main cortical hub) of the ventral stream that primarily supports auditory comprehension as well as part of MNS; (2) BA22 has a significant representation of oxytocin and dopaminergic elements; (3) it has a prominent L3 and L5 with a high density of D2 receptors, which presumably play an essential role in mu suppression.

Furthermore, CP3, CP4, C5, and C6 may merit special consideration, specifically given that these electrodes are adjacent to C3 and C4, the classical mu rhythm recording sites. And also taking into consideration that the BA40 on which these electrodes are projected refers to key regions of MNS. However, we suggest identifying these sites as the locations of the second line, given that the BA40 has lower priority on speech comprehension and less-defined L3 and L5 than BA22.

**Figure 1:**
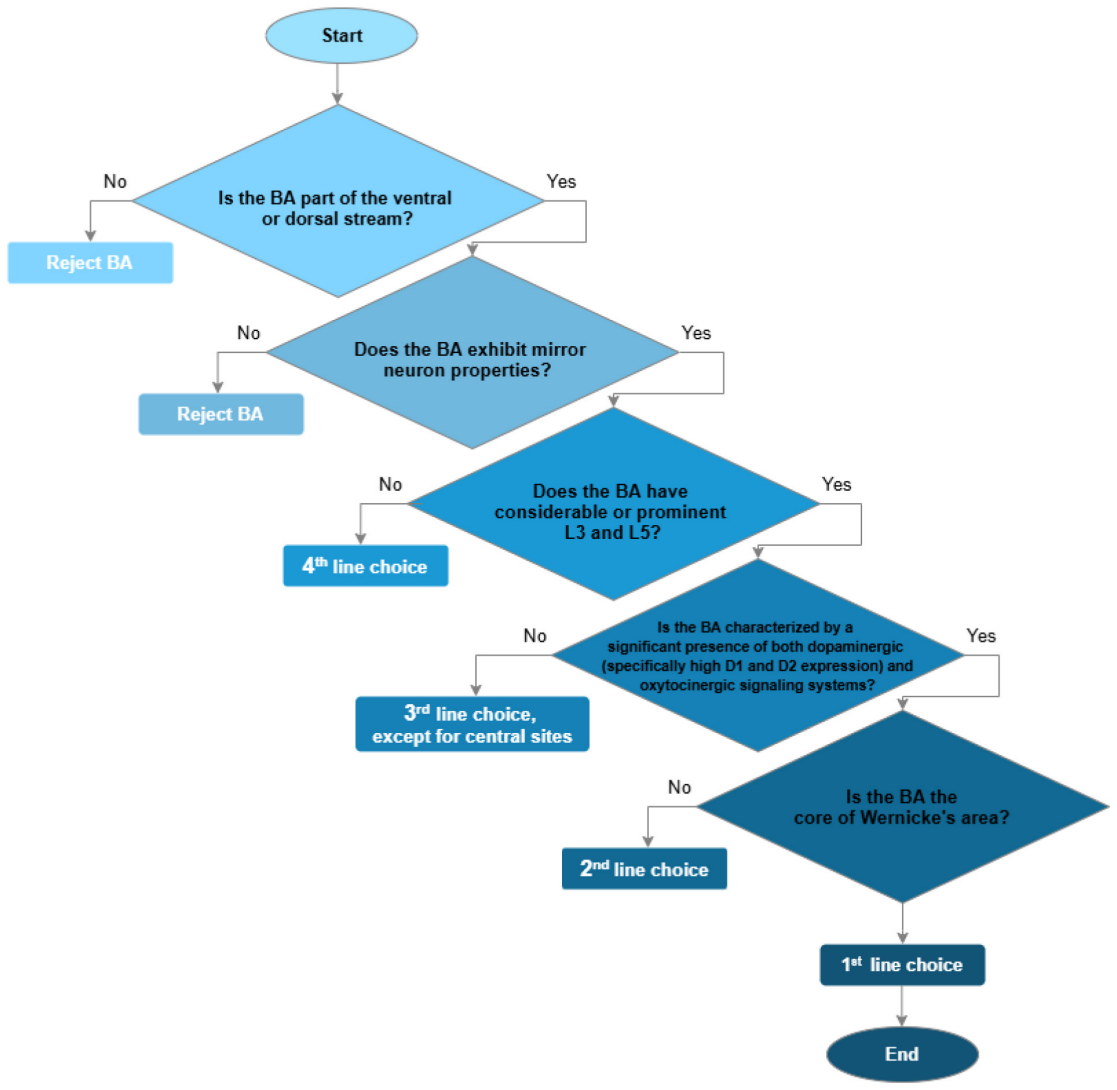
A decision flowchart summarizing the stages of justification of the choice of projection zones (BAs) of EEG electrodes (for more details see the text).

Other electrodes can be subjectively distributed as follows: P3, P4, P5, P6, TP7, TP8, FT10 represent a third line, and AF3, AF4, AF6, AFz, F3, F4, F5, F6, F7, and F8 a fourth line. The designation of frontal electrodes as locations of last-line choice is dictated by the fact that BAs where they are projected (i.e., areas 9 and 46) are primarily associated with higher-order executive functions (e.g., working memory, cognitive control) that support language comprehension indirectly. The logic underlying the formulation of electrodes' selection lines can be represented as a flowchart (Figure 1).

Thus, we share the view discussed earlier (for instance, see Saltuklaroglu et al., 2018) that electrode selection for measurements of mu activity should be determined primarily by the tasks' specificity. And selecting regions of interest should be based on the functions of cortical areas to a larger extent than just a correspondence of areas with mu oscillations.

On a final note, several important points should be mentioned. First, the use of EEG electrodes on the perimeter (for instance, FT10) is questionable, as they are easily contaminated by motor artifacts. Second, even for a specific experimental task, the use of a large electrode set is advisable.

## Limitations and future work

The proposed strategy is purely theoretical and requires empirical validation. The main interest is the direct validation of the recommended electrode sites, specifically the first-line set, in comparison with classical central locations (C3, C4, and Cz) as the most commonly used for detecting mu activity. Use of simultaneous EEG-fMRI suggests a promising trajectory for future research, as it would enable the correlation of hemodynamic activity within the ventral and dorsal streams with the topographic distribution of mu-rhythm power over the proposed sites within speech comprehension paradigms.

The use of biological extrapolations in this study is a crucial limitation that necessitates caution, as it may significantly reduce the precision and potential applicability of our conclusions. For instance, distribution patterns of neurotransmitter receptors and laminar data substantially come from primary and prefrontal regions; their extrapolation to the temporal and parietal association cortices in humans is thus approximate.
